# Supplementary material for: Morphology and development of a novel murine skeletal dysplasia
Source: PeerJ. 2019 Jul 4;7:e7180. doi: 10.7717/peerj.7180 (PMC6612423; doi:10.7717/peerj.7180)
Supplement: Table S1 — Standard error presented in parentheses. Bold font and asterisk denote statistically significant differences in group means (p < 0.05). Degree of freedom (Df), F test (F) and p-value (p). [file peerj-07-7180-s001.docx]

**Table S1.** Mean of body mass and least square means of measurements taken using µCT. Standard error presented in parenthesis. Red font and asterisk denote significant differences in means (p < 0.05). Degree of freedom (Df), F test (F) and p-value (p).

|  | Control | *Shorty* | Statistics ANCOVA |
| --- | --- | --- | --- |
| *Postnatal age P0* | | | |
| Sample size | 5 | 5 |  |
| Body mass (g) | 1.57 (0.15) | 1.50 (0.11) |  |
| Clavicle (mm) | 2.80 (0.08) | 2.91 (0.05) | Df=1 ; F=1.61 ; p=0.342 |
| Femur (mm) | **2.32 (0.09)** | **1.66 (0.06)*** | Df=1 ; F=34.848 ; p=0.04 |
| Humerus (mm) | **2.98 (0.09)** | **2.00 (0.06)*** | Df=1 ; F=76.345 ; p=0.001 |
| Scapula (mm) | **2.93 (0.07)** | **2.17 (0.05)*** | Df=1; F=71.624 ; p=0.001 |
| Tibia (mm) | **2.91 (0.15)** | **2.38 (0.09)*** | Df=1 ; F=8.873 ; p=0.41 |
| Ulna (mm) | **3.18 (0.08)** | **2.54 (0.05)*** | Df=1; F=41.830 ; p=0.003 |
| *Postnatal age P3* | | | |
| Sample size | 5 | 5 |  |
| Body mass (g) | 2.71 (0.45) | 2.41 (0.26) |  |
| Clavicle (mm) | 3.63 (0.13) | 3.46 (0.13) | Df=1 ; F=0.707 ; p=0.439 |
| Femur (mm) | **3.31 (0.11)** | **2.22 (0.11)*** | Df=1 ; F=48.916 ; p=0.001 |
| Humerus (mm) | **3.93 (0.12)** | **2.70 (0.12)*** | Df=1 ; F=49.26 ; p=0.001 |
| Scapula (mm) | **3.94 (0.13)** | **2.71 (0.13)*** | Df=1 ; F=38.797 ; p=0.002 |
| Tibia (mm) | **4.11 (0.12)** | **3.17 (0.12)*** | Df= 1; F=28.51 ; p=0.003 |
| Ulna (mm) | **4.28 (0.13)** | **3.16 (0.13)*** | Df=1; F=39.731 ; p=0.001 |
| Sample size | 4 | 4 |  |
| Skull (mm) | 11.98 (0.35) | 11.04 (0.35) | Df=1; F=3.383 ; p=0.125 |
| *Postnatal age P7* | | | |
| Sample size | 4 | 5 |  |
| Body mass (g) | 4.45 (0.75) | 4.44 (0.22) |  |
| Clavicle (mm) | 4.29 (0.18) | 4.01 (0.10) | Df=1 ; F=1.578 ; p=0.277 |
| Femur (mm) | **4.51 (0.24)** | **3.13 (0.13)*** | Df=1 ; F=20.435 ; p=0.011 |
| Humerus (mm) | **4.83 (0.09)** | **3.59 (0.05)*** | Df=1 ; F=125.132 ; p<0.001 |
| Scapula (mm) | **5.62 (0.03)** | **3.75 (0.02)*** | Df=1 ; F=1878.038 ; p<0.001 |
| Tibia (mm) | **5.86 (0.19)** | **4.44 (0.10)*** | Df=1 ; F=34.187 ; p=0.004 |
| Ulna (mm) | **5.94 (0.16)** | **4.27 (0.09)*** | Df=1 ; F=70.090 ; p=0.001 |
| Skull (mm) | **12.66 (0.39)** | **14.93 (0.21)*** | Df=1 ; F=20.166 ; p=0.011 |
| Metacarpal (mm) | 1.11 (0.12) | 1.01 (0.07) | Df=1 ; F=0.408 ; p=0.558 |
| Metatarsal (mm) | 1.69 (0.245) | 1.55 (0.13) | Df=1 ; F=0.198 ; p=0.679 |
| *Postnatal age P14* | | | |
| Sample size | 5 | 5 |  |
| Body mass (g) | 9.20 (1.86) | 6.12 (0.64) |  |
| Clavicle (mm) | 5.12 (0.07) | 4.92 (0.06) | Df=1 ; F=3.099 ; p=0.129 |
| Femur (mm) | 6.79 (0.18) | 4.48 (0.15)* | Df=1 ; F=65.276 ; p<0.001 |
| Humerus (mm) | 7.00 (0.18) | 5.05 (0.15)* | Df=1 ; F= 47.00 ; p<0.001 |
| Scapula (mm) | 7.71 (0.21) | 5.53 (0.18)* | Df=1 ; F=42.660 ; p=0.001 |
| Tibia (mm) | 9.44 (0.27) | 6.55 (0.23)* | Df=1 ; F=46.746 ; p<0.001 |
| Ulna (mm) | 8.80 (0.29) | 6.27 (0.24)* | Df=1 ; F=31.058 ; p=0.001 |
| Skull (mm) | 18.72 (0.40) | 17.69 (0.34) | Df=1 ; F=2.649 ; p=0.155 |
| Metacarpal (mm) | 1.57 (0.14) | 1.42 (0.12) | Df=1 ; F=0.428 ; p=0.537 |
| Metatarsal (mm) | 4.21 (0.31) | 3.44 (0.26) | Df=1 ; F=2.516 ; p=0.164 |
| *Postnatal age P21* | | | |
| Sample size | 5 | 4 |  |
| Body mass (g) | 14.60 (2.13) | 10.16 (2.05) |  |
| Clavicle (mm) | 5.63 (0.28) | 5.97 (0.32) | Df=1 ; F=0.463 ; p=0.521 |
| Femur (mm) | **9.97 (0.14)** | **7.77 (0.16)*** | Df=1 ; F=78.391 ; p<0.001 |
| Humerus (mm) | **7.75 (0.14)** | **5.84 (0.16)*** | Df=1 ; F=58.572 ; p<0.001 |
| Scapula (mm) | **8.50 (0.25)** | **6.87 (0.29)*** | Df=1 ; F=12.767 ; p=0.012 |
| Tibia (mm) | **10.77 (0.29)** | **9.10 (0.34)*** | Df=1 ; F=10.116 ; p=0.019 |
| Ulna (mm) | **9.60 (0.10)** | **7.84 (0.12)*** | Df=1 ; F=89.463 ; p<0.001 |
| Skull (mm) | 20.05 (0.14) | 20.28 (0.17) | Df=1 ; F=0.783 ; p=0.410 |
| Metacarpal (mm) | 1.62 (0.07) | 1.85 (0.08) | Df=1 ; F=3.285 ; p=0.120 |
| Metatarsal (mm) | 4.59 (0.12) | 5.01 (0.14) | Df=1 ; F=3.848 ; p=0.097 |
| *Postnatal age P28* | | | |
| Sample size | 5 | 5 |  |
| Body mass (g) | 25.18 (3.94) | 14.67 (2.27) |  |
| Clavicle (mm) | 6.42 (0.17) | 6.70 (0.17) | Df=1 ; F=0.780 ; p=0.407 |
| Femur (mm) | **11.88 (0.16)** | **9.45 (0.16)*** | Df=1 ; F=74.572 ; p<0.001 |
| Humerus (mm) | **8.61 (0.20)** | **6.00 (0.20)*** | Df=1 ; F=53.266 ; p<0.001 |
| Scapula (mm) | 9.13 (0.44) | 7.53 (0.44) | Df=1 ; F=4.017 ; p=0.085 |
| Tibia (mm) | **13.75 (0.17)** | **10.18 (0.17)*** | Df=1 ; F=128.872 ; p<0.001 |
| Ulna (mm) | **11.29 (0.24)** | **8.87 (0.24)*** | Df=1 ; F=32.296 ; p=0.001 |
| Skull (mm) | 21.31 (0.20) | 20.90 (0.20) | Df=1 ; F=1.334 ; p=0.286 |
| Metacarpal (mm) | **1.65 (0.08)** | **2.11 (0.08)*** | Df=1 ; F=9.190 ; p=0.19 |
| Metatarsal (mm) | 5.50 (0.09) | 5.23 (0.09) | Df=1 ; F=2.384 ; p=0.166 |
| *Postnatal age P42* | | | |
| Sample size | 5 | 5 |  |
| Body mass (g) | 27.19 (3.88) | 19.19 (4.13) |  |
| Clavicle (mm) | 7.22 (0.16) | 7.46 (0.12) | Df=1 ; F=1.072 ; p=0.348 |
| Femur (mm) | **13.63 (0.21)** | **10.64 (0.15)*** | Df=1 ; F=97.496 ; p<0.001 |
| Humerus (mm) | **9.56 (0.17)** | **7.08 (0.12)*** | Df=1 ; F=106.275 ; p<0.001 |
| Scapula (mm) | **11.01 (0.24)** | **8.33 (0.17)*** | Df=1 ; F=61.134 ; p=0.001 |
| Tibia (mm) | **15.28 (0.39)** | **10.92 (0.27)*** | Df=1 ; F=62.887 ; p=0.001 |
| Ulna (mm) | **13.10 (0.52)** | **10.17 (0.37)*** | Df=1 ; F=15.824 ; p=0.011 |
| Skull (mm) | **21.84 (0.09)** | **22.59 (0.06)*** | Df=1 ; F=32.795 ; p=0.002 |
| Metacarpal (mm) | 1.89 (0.15) | 1.96 (0.11) | Df=1 ; F=0.97 ; p=0.768 |
| Metatarsal (mm) | 5.49 (0.09( | 5.70 (0.06) | Df=1 ; F2.523= ; p=0.173 |
| *Postnatal age P75* | | | |
| Sample size | 5 | 4 |  |
| Body mass (g) | 35.17 (6.32) | 28.18 (3.86) |  |
| Clavicle (mm) | 8.25 (0.20) | 8.27 (0.17) | Df=1 ; F=0.004 ; p=0.951 |
| Femur (mm) | **16.34 (0.24)** | **11.98 (0.20)*** | Df=1 ; F=154.206 ; p<0.001 |
| Humerus (mm) | **11.37 (0.13)** | **7.98 (0.11)*** | Df=1 ; F=328.176 ; p<0.001 |
| Scapula (mm) | **11.74 (0.31)** | **9.64 (0.26)*** | Df=1 ; F=21.214 ; p=0.010 |
| Tibia (mm) | **17.52 (0.32)** | **12.25 (0.27)*** | Df=1 ; F=127.206 ; p<0.001 |
| Ulna (mm) | **14.70 (0.31)** | **11.01 (0.25)*** | Df=1 ; F=69.142 ; p=0.001 |
| Skull (mm) | 24.27 (0.31) | 23.44 (0.26) | Df=1 ; F=3.443 ; p=0.137 |
| Metacarpal (mm) | 1.86 (0.07) | 1.97 (0.06) | Df=1 ; F=1.255 ; p0.325 |
| Metatarsal (mm) | **6.44 (0.09)** | **5.99 (0.07)*** | Df=1 ; F=12.147 ; p=0.025 |
| *Postnatal age P100* | | | |
| Sample size | 5 | 5 |  |
| Body mass (g) | 43.58 (11.62) | 30.50 (5.88) |  |
| Clavicle (mm) | 8.45 (0.14) | 8.06 (0.13) | Df=1 ; F=3.305 ; p=0.119 |
| Femur (mm) | **16.79 (0.17)** | **12.34 (0.15)*** | Df=1 ; F=314.575 ; p<0.001 |
| Humerus (mm) | **11.69 (0.16)** | **8.22 (0.14)*** | Df=1 ; F=216.879 ; p<0.001 |
| Scapula (mm) | **12.73 (0.25)** | **9.56 (0.22)*** | Df=1 ; F=72.629 ; p<0.001 |
| Tibia (mm) | **17.92 (0.17)** | **13.40 (0.15)*** | Df=1 ; F=326.417 ; p<0.001 |
| Ulna (mm) | **15.25 (0.45)** | **11.38 (0.39)*** | Df=1 ; F=33.089 ; p=0.001 |
| Skull (mm) | **24.92 (0.28)** | **23.71 (0.24)*** | Df=1 ; F=8.562 ; p=0.026 |
| Metacarpal (mm) | 1.87 (0.05) | 1.91 (0.04) | Df=1 ; F=0.316 ; p=0.594 |
| Metatarsal (mm) | **6.24 (0.10)** | **5.77 (0.09)*** | Df=1 ; F=9.414 ; p=0.022 |
| *Postnatal age P125* | | | |
| Sample size | 5 | 4 |  |
| Body mass (g) | 39.50 (2.90) | 27.97 (3.16) |  |
| Clavicle (mm) | 8.03 (0.25) | 8.46 (0.31) | Df=1 ; F=0.673 ; p=0.44 |
| Femur (mm) | **16.35 (0.23)** | **13.56 (0.28)*** | Df=1 ; F=34.046 ; p=0.001 |
| Humerus (mm) | **11.26 (0.19)** | **9.07 (0.32)*** | Df=1 ; F=16.090 ; p=0.007 |
| Scapula (mm) | **12.63 (0.31)** | **10.25 (0.37)*** | Df=1 ; F=14.469 ; p=0.009 |
| Tibia (mm) | **17.85 (0.34)** | **13.61 (0.41)*** | Df=1 ; F=36.973 ; p=0.001 |
| Ulna (mm) | **14.91 (0.48)** | **11.89 (0.57)*** | Df=1 ; F=9.720 ; p=0.021 |
| Skull (mm) | 24.89 (0.36) | 24.16 (0.44) | Df=1 ; F=0.984 ; p=0.359 |
| Metacarpal (mm) | **20.36 (0.05)** | **17.14 (0.06)*** | Df=1 ; F=8.993 ; p=0.024 |
| Metatarsal (mm) | 6.18 (0.11) | 5.93 (0.14) | Df=1 ; F=1.233 ; p=0.309 |
