# Supplementary material for: Morphology and development of a novel murine skeletal dysplasia
Source: PeerJ. 2019 Jul 4;7:e7180. doi: 10.7717/peerj.7180 (PMC6612423; doi:10.7717/peerj.7180)
Supplement: Table S2 — AD indicates autosomal dominant, AR indicate autosomal recessive. EO indicates that the dysplasia interferes with endochondral ossification, while IO indicates that the dysplasia interferes with intramembranous ossification. [file peerj-07-7180-s002.docx]

**Table S2.** Subset of non-lethal skeletal dysplasia. AD indicates autosomal dominant, AR indicate autosomal recessive. EO indicates that the dysplasia interferes with endochondral ossification, while IO indicates that the dysplasia interferes with intramembranous ossification.

| Disorder | Inheritance | Gene | Ossification | Characteristic |
| --- | --- | --- | --- | --- |
| Achondroplasia | AD | *FGFR3* | EO; IO | Expansion growth plate, change in skull bones (nasal and frontal)^1^ |
| Pseudoachondroplasia | AD | *COMP* | EO | Growth plate disorganized, reduce PC and increased apoptosis^2^ |
| Rhizomelic chondrodysplasia punctata | AR | *PEX7* | EO; IO | Neurological impairment, cataracts, facial dysmorphys^3^ |
| Spondylo-epiphyseal dysplasia | AD | *COL2A1* | EO; IO | Short limbs and trunk. Scoliosis. Early arthritis. Cleft palate^4^ |
| Metaphyseal chondrodysplasia Jansen-type | AD | *PTHR1* | EO; IO | Severe. Hindlimbs short and angulated  Spaced and prominent eyes. High skull vault^5^ |
| Metaphyseal chondrodysplasia Schmid-type | AD | *COL10A1* | EO | Skeletal changes appear during postnatal development. Short stature^4^ |
| Hypophosphatasia | AR or AD | *TNSALP* | EO | Short and bowed limbs and dental disease. Hypomineralization and diaphyseal spur^6^ |
| Diastrophic dysplasia | AR | *SLC26A2* | EO; IO | Angulated thumbs, scoliosis, sometimes cleft palate^7^ |
| Campomelic dysplasia | AD | *SOX9* | EO; IO | Short bowed limbs, and short hand and feet. Head facial deformities^8,9^ |
| Pallister-Hall syndrome | AD | *GLI3* | EO; IO | Polydactyly, Bifid epiglottis, flat nasal bridge^9,10^ Biesecker, Krakow |

1 Wang, Y. *et al.* A mouse model for achondroplasia produced by targeting fibroblast growth factor receptor 3. *Proceedings of the National Academy of Sciences* **96**, 4455-4460 (1999).

2 Posey, K. L. *et al.* Chondrocyte‐Specific Pathology During Skeletal Growth and Therapeutics in a Murine Model of Pseudoachondroplasia. *Journal of Bone and Mineral Research* **29**, 1258-1268 (2014).

3 White, A. L., Modaff, P., Holland-Morris, F. & Pauli, R. M. Natural history of rhizomelic chondrodysplasia punctata. **118A**, 332-342 (2003).

4 Shah, I. P., Varghese, B. & Fernandes, J. A. Skeletal dysplasia. *Surgery (Oxford)* (2016).

5 Silve, D. C. & Jüppner, D. H. Jansen’s metaphyseal chondrodysplasia. (2005).

6 Wenkert, D. *et al.* Hypophosphatasia: nonlethal disease despite skeletal presentation in utero (17 new cases and literature review). *Journal of Bone and Mineral Research* **26**, 2389-2398 (2011).

7 Hurst, J. A., Firth, H. V. & Smithson, S. Skeletal dysplasias. *Fetal and Neonatal Genetics* **10**, 233-241 (2005).

8 Mansour, S. *et al.* The phenotype of survivors of campomelic dysplasia. *Journal of Medical Genetics* **39**, 597 (2002).

9 Krakow, D. & Rimoin, D. L. The skeletal dysplasias. **12**, 327-341 (2010).

10 Biesecker, L. G. & Graham, J. Pallister-Hall syndrome. *Journal of medical genetics* **33**, 585-589 (1996).
