# Supplementary figures and images for: Morphology and development of a novel murine skeletal dysplasia
Source: PeerJ. 2019 Jul 4;7:e7180. doi: 10.7717/peerj.7180 (PMC6612423; doi:10.7717/peerj.7180)

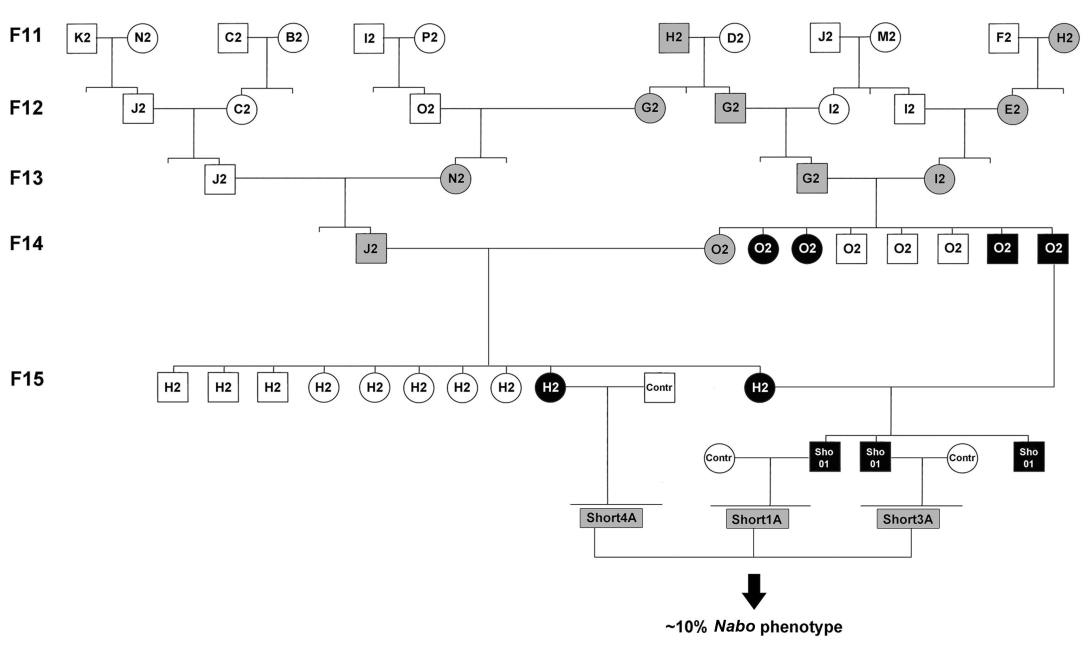

Supplement: Figure S1 — F indicates the generation of selection in the Longshanks artificial selection experiment. Squares indicate males, circles indicate females. The family name is indicated inside the squares and circles. The lines between the circles and squares represent breeding pairs and subsequent progeny. Black circles and squares indicate Nabo phenotype. Grey circles and squares are hypothesized heterozygotes. In F11, H2 male and female are siblings. [file peerj-07-7180-s003.png]

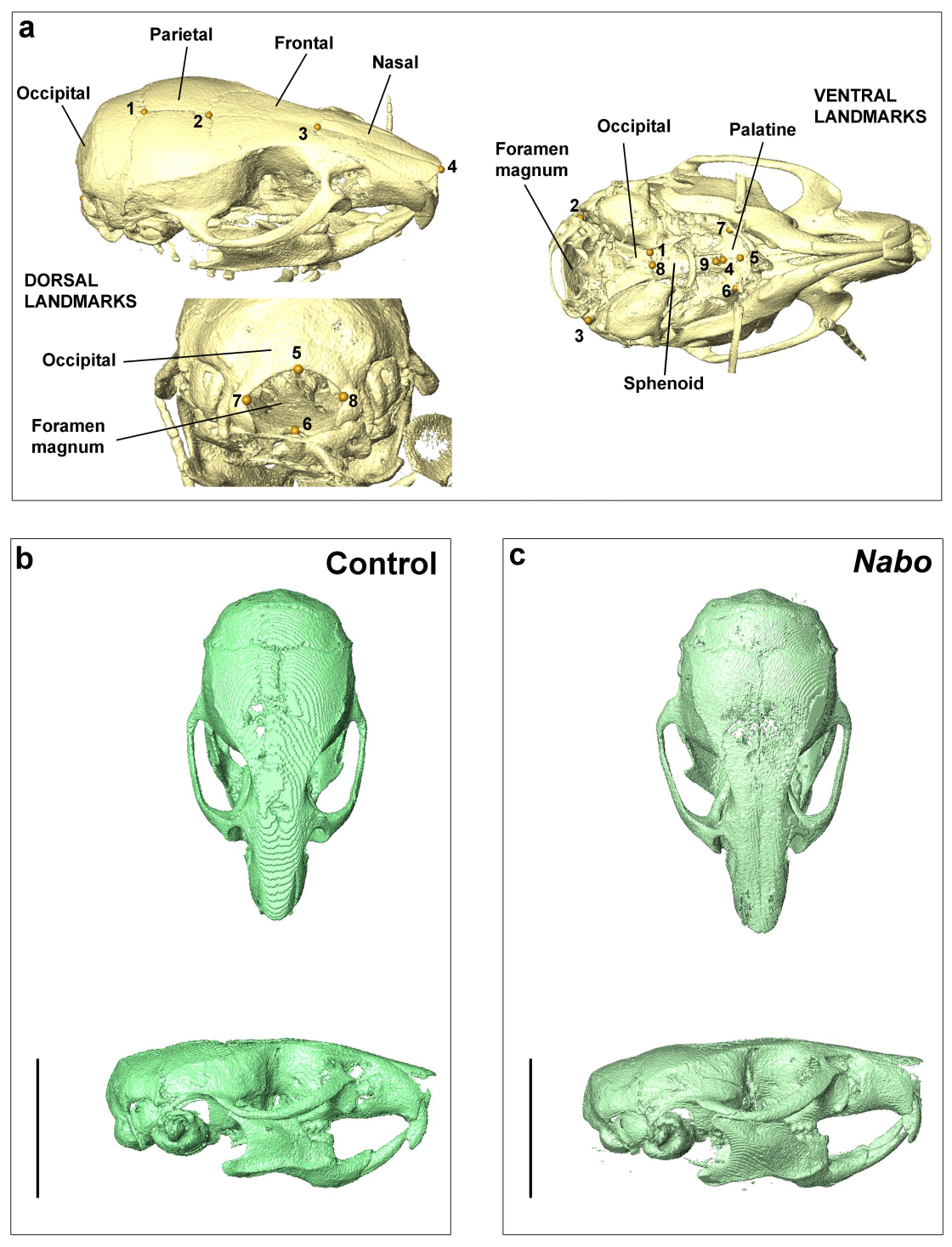

Supplement: Figure S2 — a. Representation of the landmarks placed in the dorsal and ventral elements of the skull using Amira. b.c. Dorsal and lateral view of a wildtype (b) and Nabo (c) specimen. Scale bar = 10 mm. [file peerj-07-7180-s004.png]

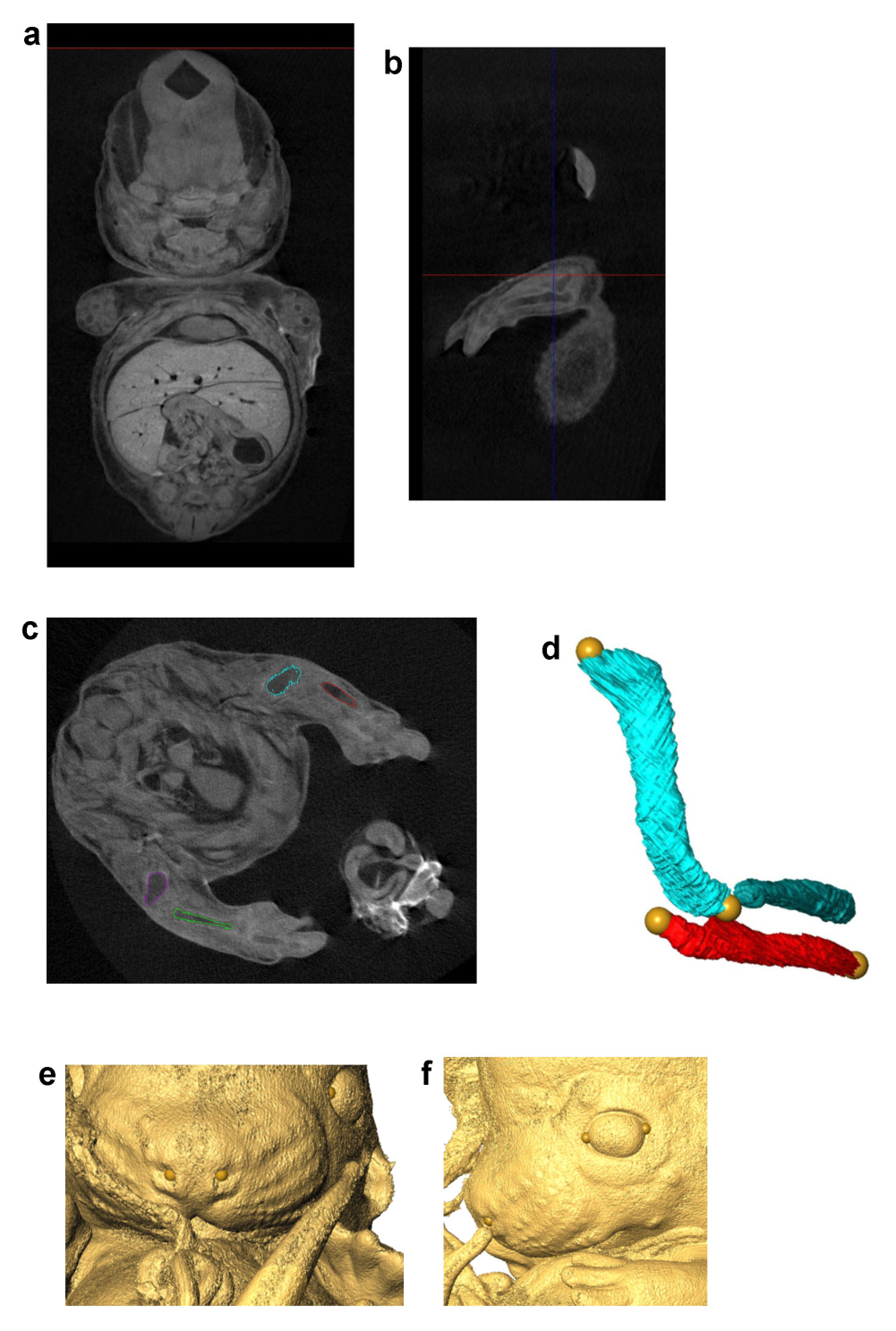

Supplement: Figure S3 — a.b.c. E14 embryo stained with Lugol’s iodine, in coronal (a), parasagittal (b) and transverse (c) section from µCT. Outlines in c indicate limb cartilages. d. 3D render of forelimb with humerus in cyan, radius in green and ulna in red. 3D-landmarks used for linear measurements. e.f. 3D surface rendering of E14 embryo stained with Lugol’s iodine. 3D-landmarks in the nose (e) and eye (f) were used to derive internasal and interorbital distances, respectively, as covariates. [file peerj-07-7180-s005.png]

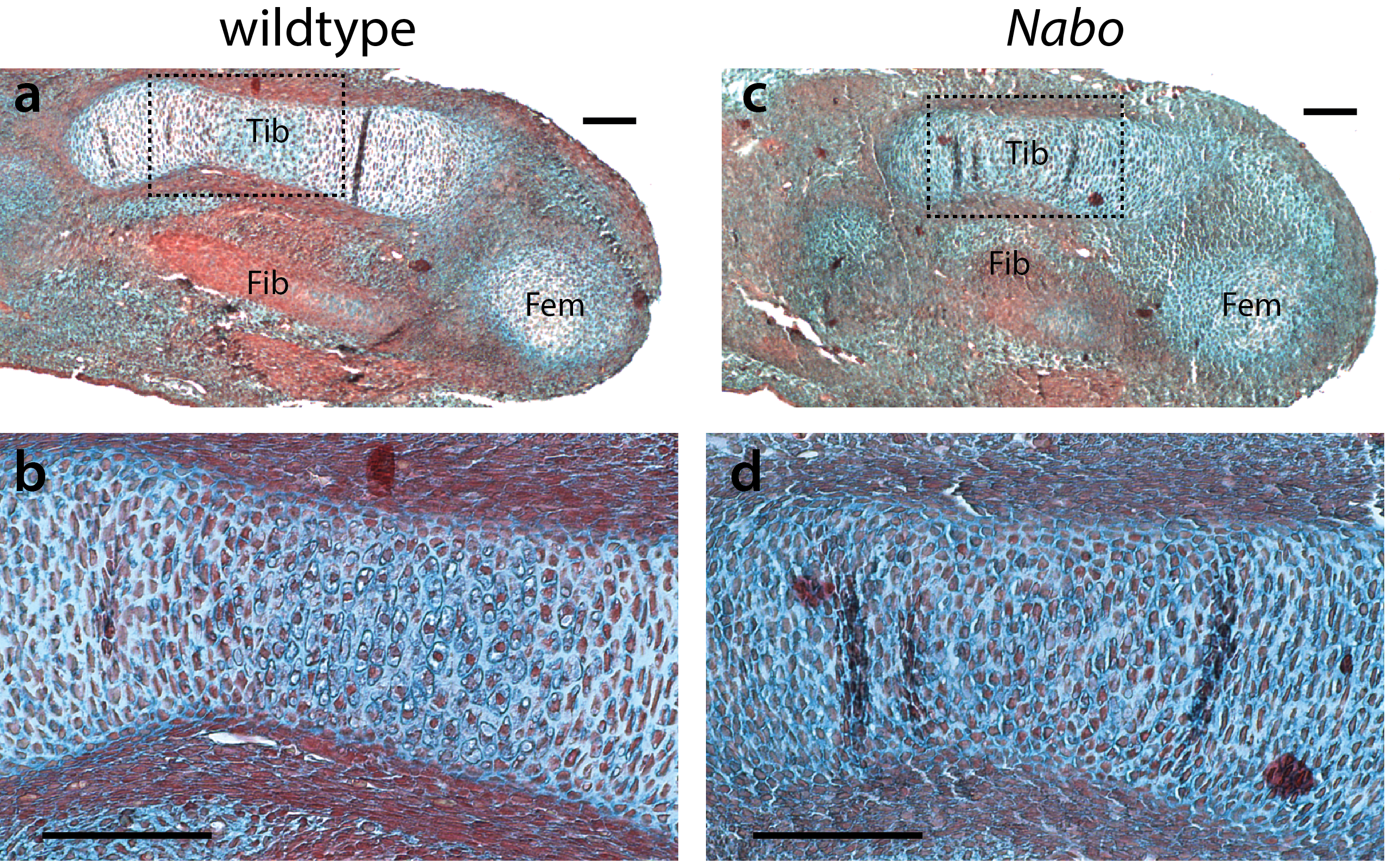

Supplement: Figure S4 — Histology of tibia anlagen at embryonic stage E14.5. (a,c) Tibia anlagen (Tib) is shorter in Nabo (c) compared to wildtype (a). Portions of the fibula (Fib) and femur (Fem) anlagen are also visible. (b,d) histological features of tibia anlagen (magnified region indicated by dashed boxes in a,c) showing fewer hypertrophic chondrocytes in Nabo (d). Scale bar = 100µm. [file peerj-07-7180-s006.png]
